# Supplementary material for: Cross-protection and cross-feeding between Klebsiella pneumoniae and Acinetobacter baumannii promotes their co-existence
Source: Nat Commun. 2023 Feb 9;14:702. doi: 10.1038/s41467-023-36252-2 (PMC9911699; doi:10.1038/s41467-023-36252-2)
Supplement: Supplementary file 1 — Supplementary Information [file 41467_2023_36252_MOESM1_ESM.pdf]

Supplementary Figures

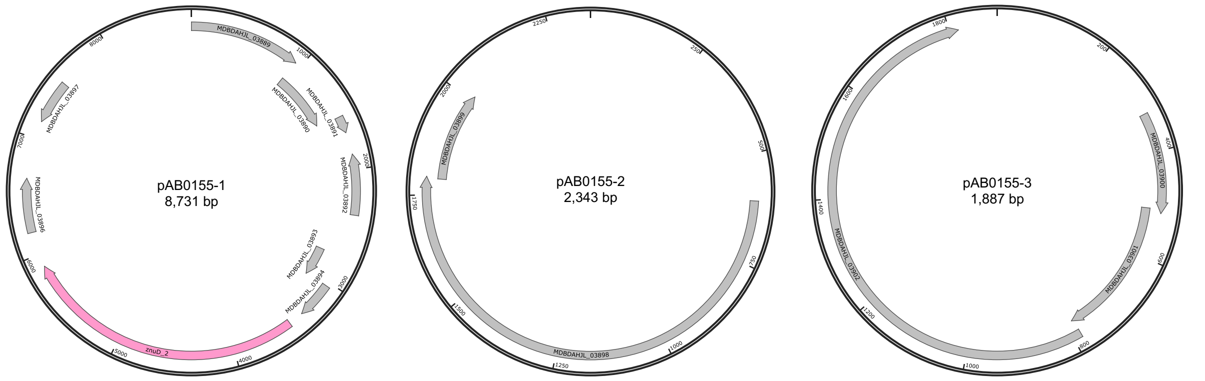

Figure S1 – AB6870155 plasmids constructed using SnapGene® software. Transporters are indicated in pink and predicted genes are in grey.

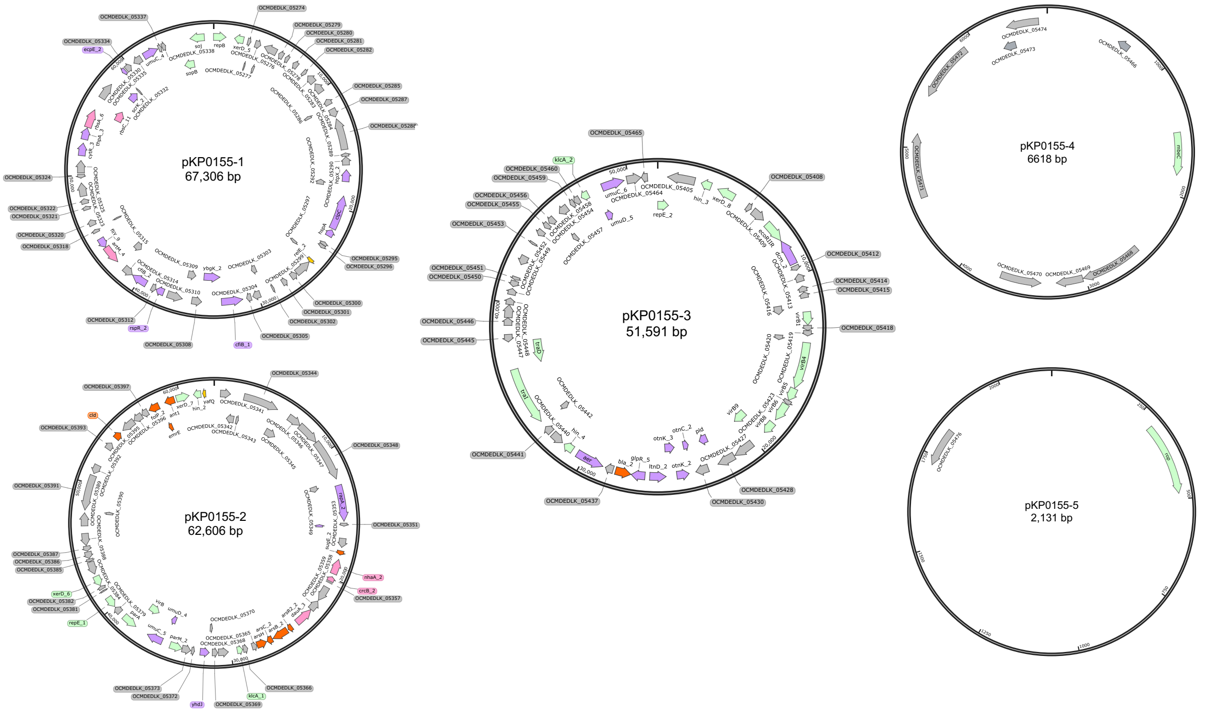

Figure S2 – KP6870155 plasmids constructed using SnapGene® software. Regions of antimicrobial resistance are orange, plasmid maintenance and mobilisation genes are in green, toxin/antitoxin genes are gold, transporters are pink, predicted genes are grey and all other genes are purple.

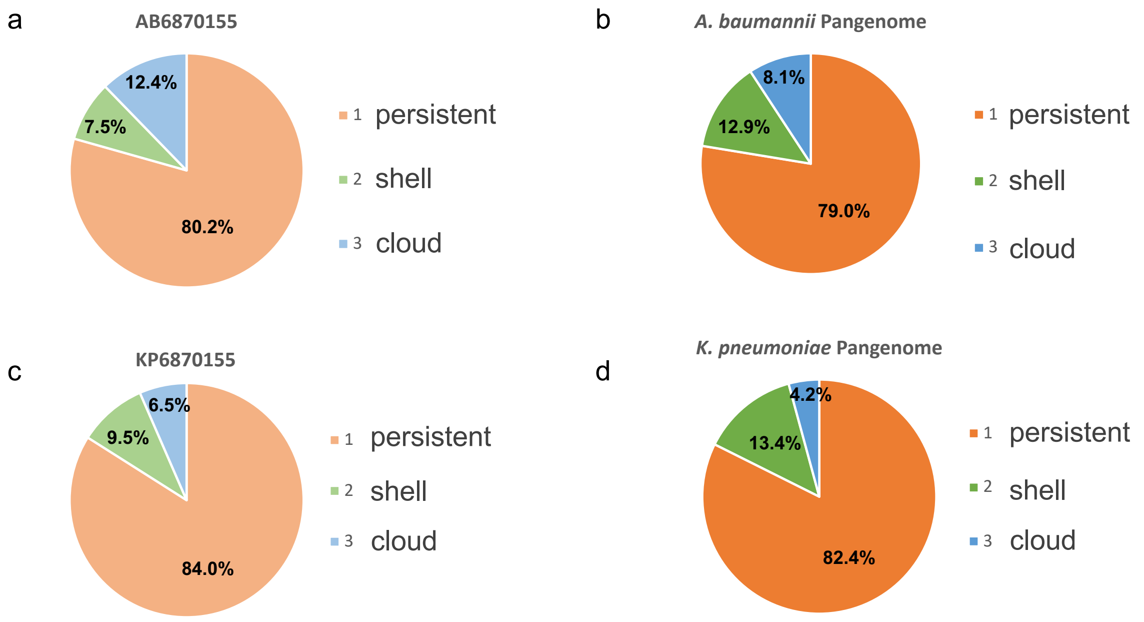

Figure S3 – Pangenomes of AB6870155 and KP6870155. Distribution of persistent (soft-core), shell (core/shell) and cloud (accessory/dispensable) genes for **a,** AB6870155 compared to **b,** pangenome containing 173 strains of *A. baumannii* and **c,** KP6870155 compared to **d,** pangenome containing 573 strains of *K. pneumoniae*. Pangenomes computed by PPanGGOLiN (20). Source data are provided as a Source Data file.

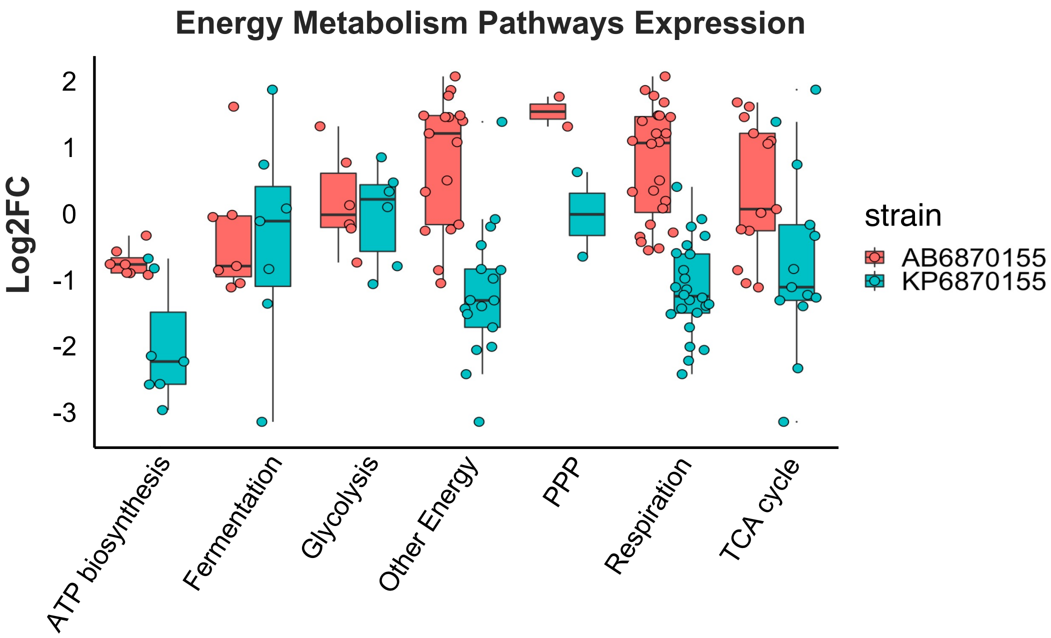

Figure S4 – RNA-seq genetic expression changes in SLMM grown co-cultures. Distribution of gene expression across energy metabolism pathways as measured by the log fold change (Log2FC) of gene expression in co-cultures of each strain versus their pure culture counterparts (n = 3 independent bacterial cultures). ATP – adenosine triphosphate, PPP – pentose phosphate pathway, TCA – tricarboxylic acid cycle. Boxes are bound by the first and

third quartile with a horizontal line at the median and whiskers represent 1.5x the interquartile range. Source data are provided as a Source Data file.

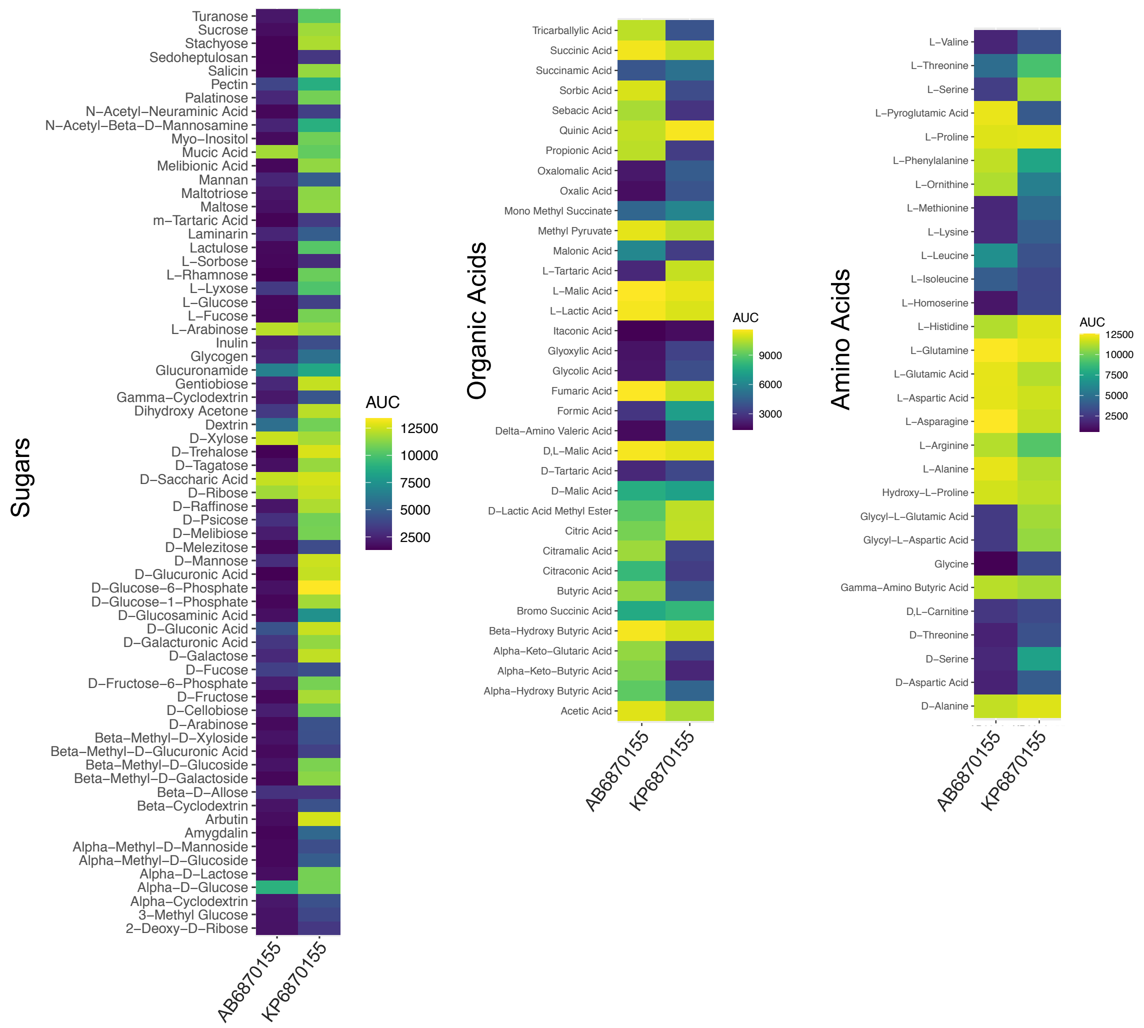

Figure S5 – Phenotypic microarrays of AB6870155 and KP6870155. AUC values in arbitrary Omnilog units measured in AB6870155 and KP6870155 grown on PM Biolog plates 1-2 for 48 hours. Source data are provided as a Source Data file.

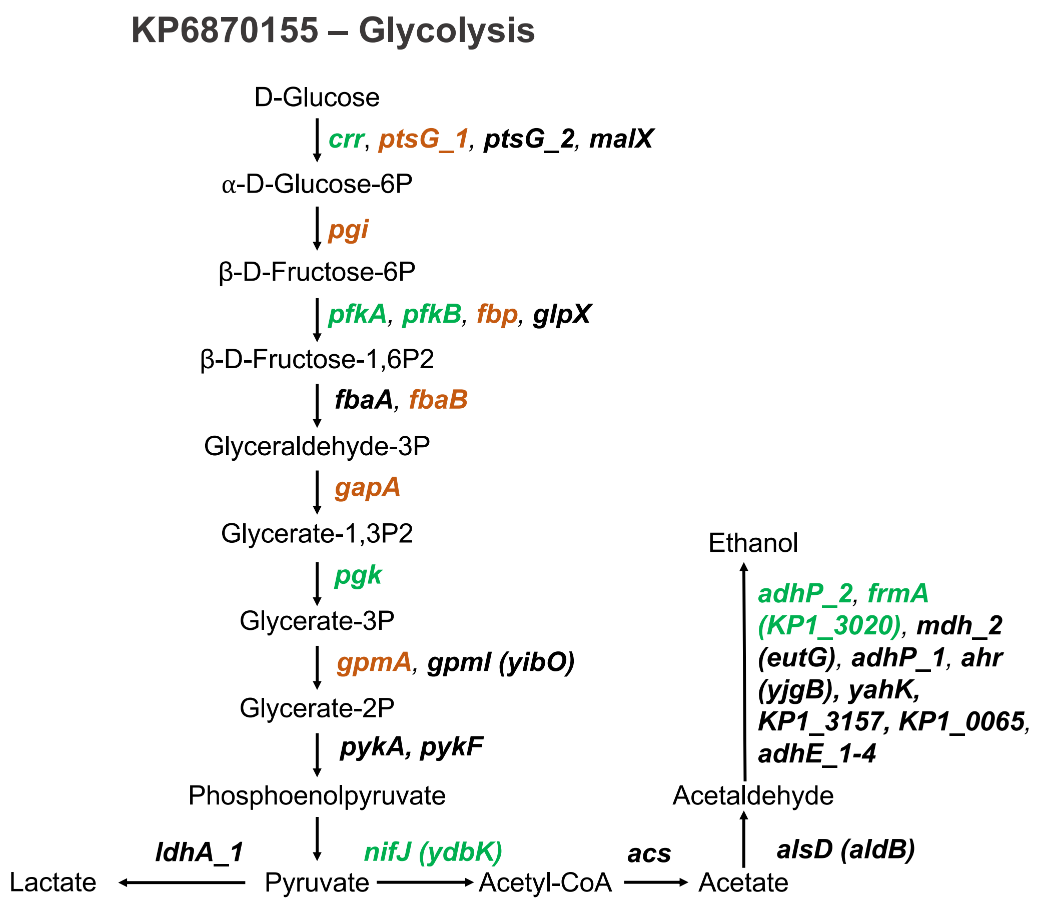

Figure S6 – Glycolysis pathway expression in *K. pneumoniae* KP6870155 grown in co-culture biofilms with *A. baumannii* AB6870155. Genes in green text represent those with a significant (adj.pvalue ≤ 0.05) increase in log 2-fold change expression when KP6870155 is grown in co-culture biofilms with AB6870155 versus mono-culture biofilms in SLMM. Those in orange text represent genes with significantly (adj.pvalue ≤ 0.05) lower transcript levels and those in black text represent those with no significant (adj.pvalue > 0.05) change in expression. Pathway and genes were obtained from the KEGG database [121,122]. Source data are provided as a Source Data file.

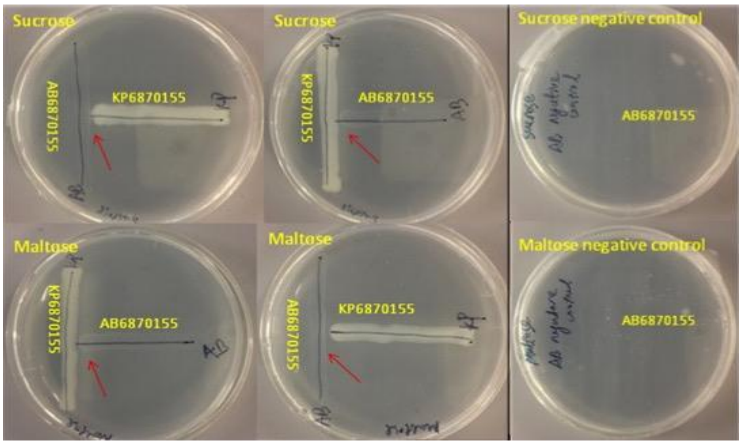

Figure S7 - Cross-feeding between AB6870155 and KP6870155. Utilization of sucrose and maltose as sole carbon source on 1.2% agar M9 minimal medium. The two strains were

inoculated adjacent to each other using a sterile loop. The media contains only a single carbon source, as indicated in yellow text. Red arrow points to the growth edge of AB6870155.

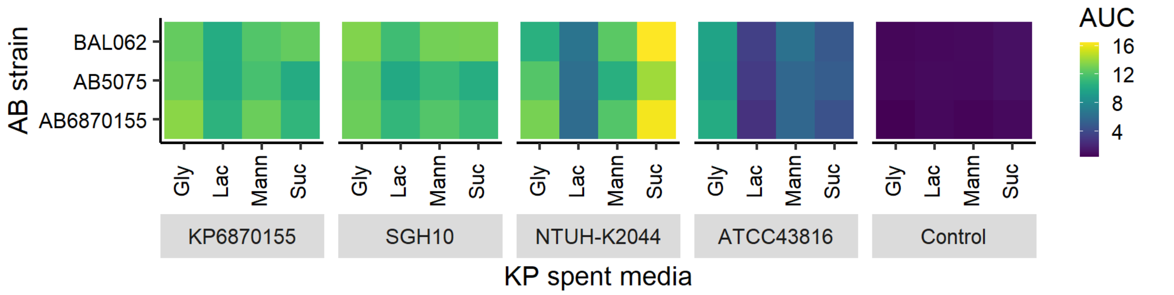

Figure S8 – Successional co-feeding of *A. baumannii* strains with spent media from various *K. pneumoniae* strains. Growth is represented by area under curve (AUC) measurements taken from growth curves of *A. baumannii* strains (BAL062, AB5075 and AB6870155) grown in filtered spent media from *K. pneumoniae* strains (KP6870155, SGH10, NTUH-K2044 and ATCC 43816) fed with defined carbon sources; Gly = glycerol, Lac = lactose, Mann = mannitol, Suc = sucrose. Source data are provided as a Source Data file.

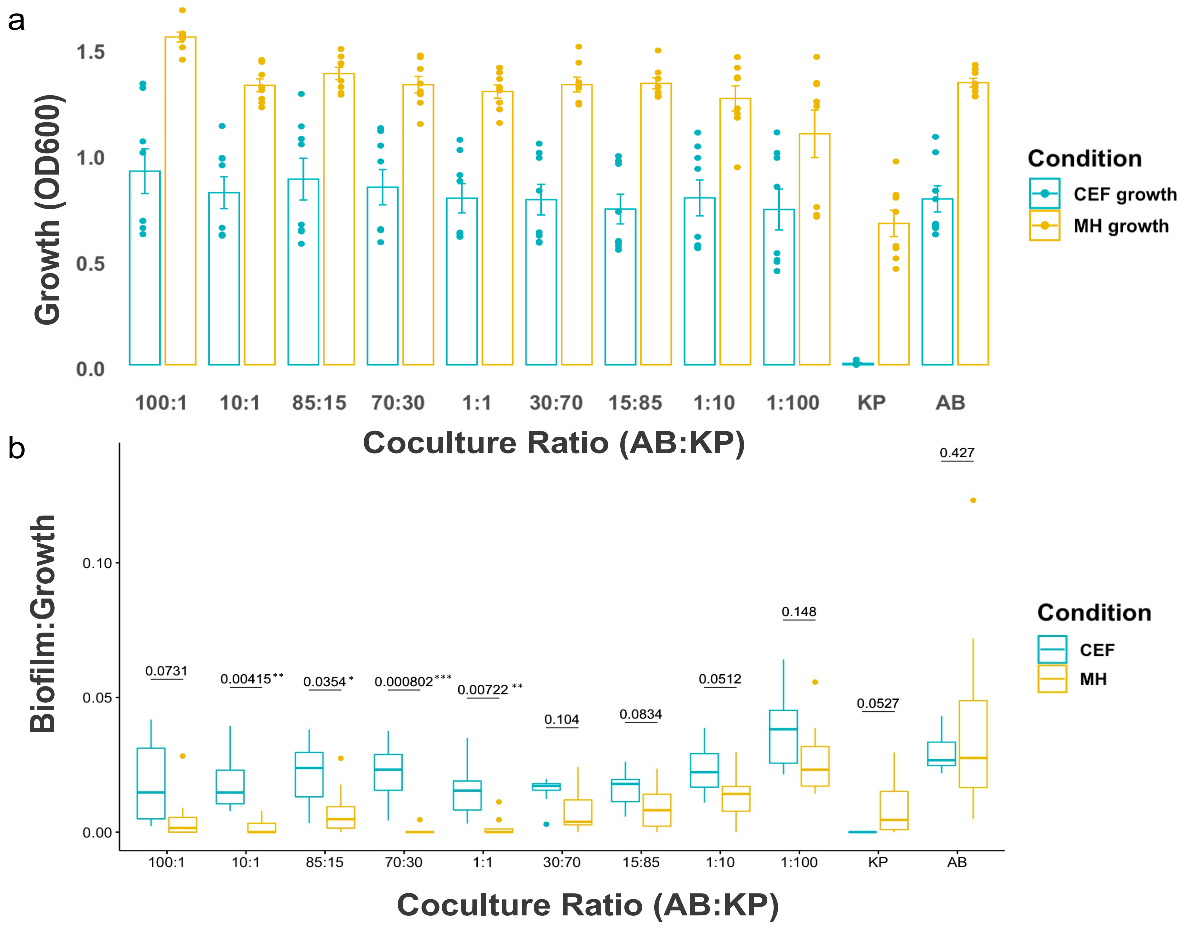

Figure S9 - Biofilm and growth in the presence and absence of cefotaxime (512 µg/mL). a, Growth measured by optical density (OD 600nm) of mono-cultures of KP6870155 (KP) and AB6870155 (AB) and co-cultures at various ratios of AB6870155:KP6870155 grown in MH broth or MH broth + 512 µg/mL cefotaxime (*n* = 8). Data are presented as mean values +/- SEM **b,** Biofilm (OD 550 nm) relative to Growth (OD 600 nm) of mono-cultures of

KP6870155 (KP) and AB6870155 (AB) and co-cultures at various ratios of AB6870155:KP6870155 grown in MH broth or MH broth + 512 µg/mL cefotaxime ($n = 8$). Boxes are bound by the first and third quartile with a horizontal line at the median and whiskers represent 1.5x the interquartile range. *P*-values were calculated with two-tailed Students t-test (*$p < 0.05$, **$p < 0.01$, ***$p < 0.001$). Source data are provided as a Source Data file.

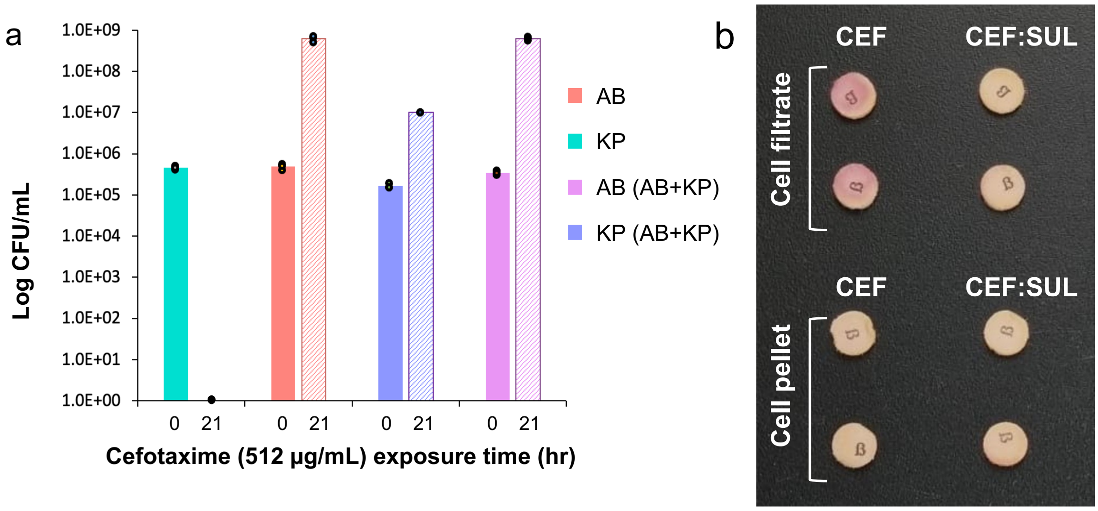

Figure S10 – Cross-protection in physically interacting and separated co-cultures of *A. baumannii* and *K. pneumoniae* and secretion of cephalosporinases. a, Survival measured by colony counts (CFU/mL) of mono-cultures, AB6870155 (AB) and KP6870155 (KP), and co-cultures of AB6870155 + KP6870155 (AB+KP) at 70:30 ratio after initial (0 hr) and 21 hr of exposure to 512 µg/mL cefotaxime ($n = 3$ independent bacterial cultures). Red colony counts corresponding to KP6870155 on MacConkey agar in the co-culture plated cells are represented by KP (AB+KP) and white colony counts corresponding to AB6870155 in the co-culture plated cells are represented by AB (AB+KP). Data are presented as mean values +/- SEM. **b,** nitrocefin discs treated with filter sterilised cell supernatant (cell filtrate) and washed cells (cell pellet) of *A. baumannii* grown in the presence of 32 µg/ml cefotaxime or 16 µg/ml cefotaxime:sulfbactam (2:1). Source data are provided as a Source Data file.

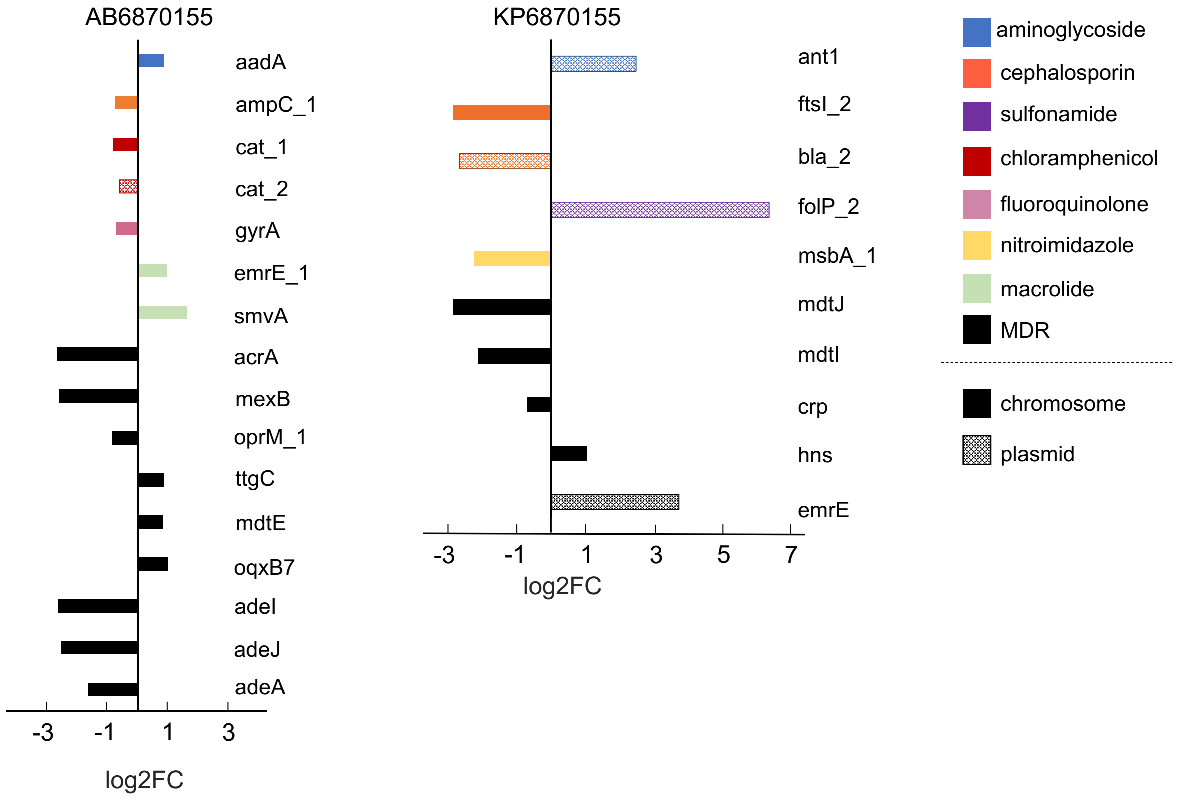

Figure S11 – Expression of antibiotic resistance genes in *A. baumannii* AB6870155 and *K. pneumoniae* KP6870155 mixed-species co-cultures. X-axis values represent the log2 fold change in gene expression of resistance genes with a minimal significance threshold (log2FC ≥ | 0.6 |, adj.pvalue ≤ 0.05, Wald Test Benjamini-Hochberg corrected) of AB6870155 co-cultured with KP6870155 versus AB6870155 mono-cultures, and KP6870155 co-cultured with AB6870155 versus KP6870155 mono-cultures. Bar colours indicate the class of antibiotic the genes confer resistance to. Solid bars indicate chromosomal genes and pattern filled bars represent genes located on plasmids. Source data are provided as a Source Data file.

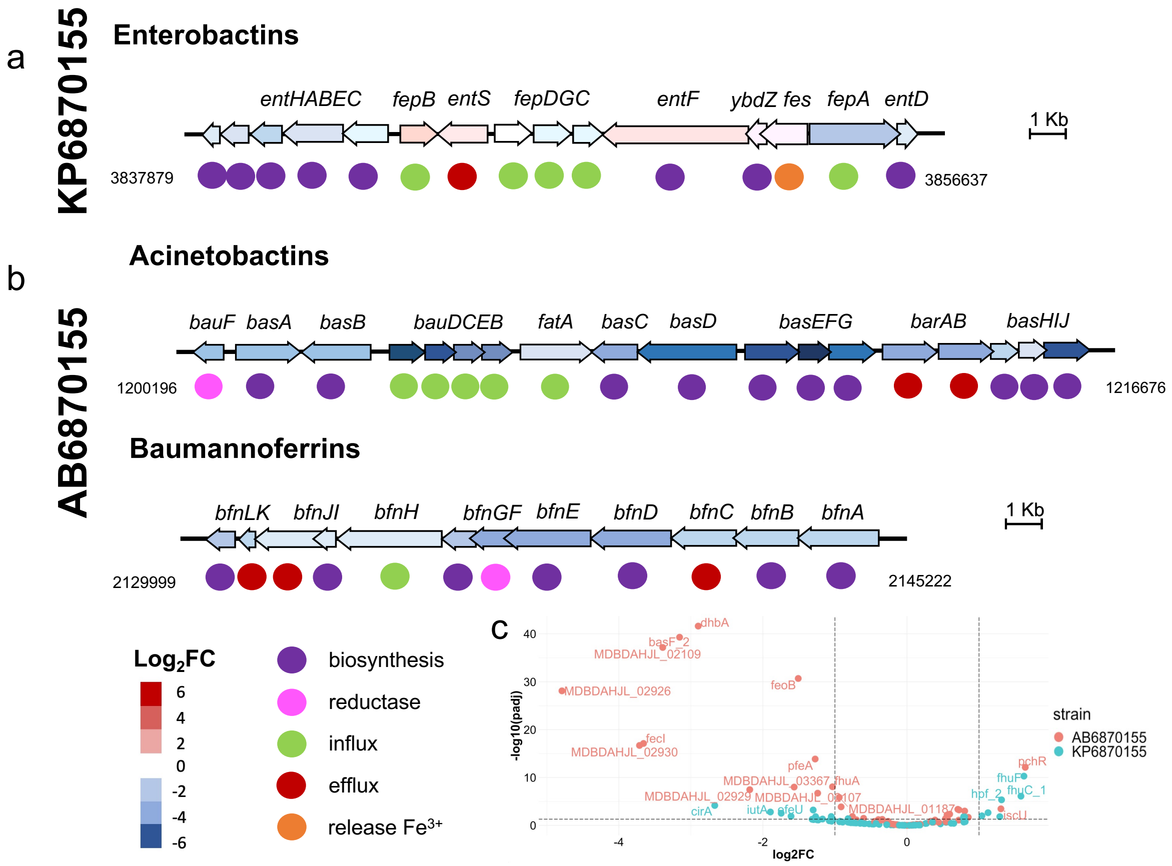

Figure S12 – Genetic analysis of siderophore loci in *K. pneumoniae* KP6870155 and *A. baumannii* AB6870155. **a,** *K. pneumoniae* KP6870155 enterobactin genetic loci, and **b,** *A. baumannii* AB6870155 acinetobactin and baumannoferrin genetic loci, with arrows representing genes and arrow colours depicting the log2 fold change gene expression of KP6870155 co-cultured with AB6870155 versus KP6870155 mono-cultures. Coloured dots indicate the functional role for each gene product. **c,** RNA-seq expression of all other iron-related genes (excluding enterobactins, acinetobactin and baumannoferrin) identified by FeGenie [108] in AB6870155 and KP6870155 co-cultures versus their respective mono-cultures. *P*-values were Wald Test Benjamini-Hochberg corrected. Source data are provided as a Source Data file.

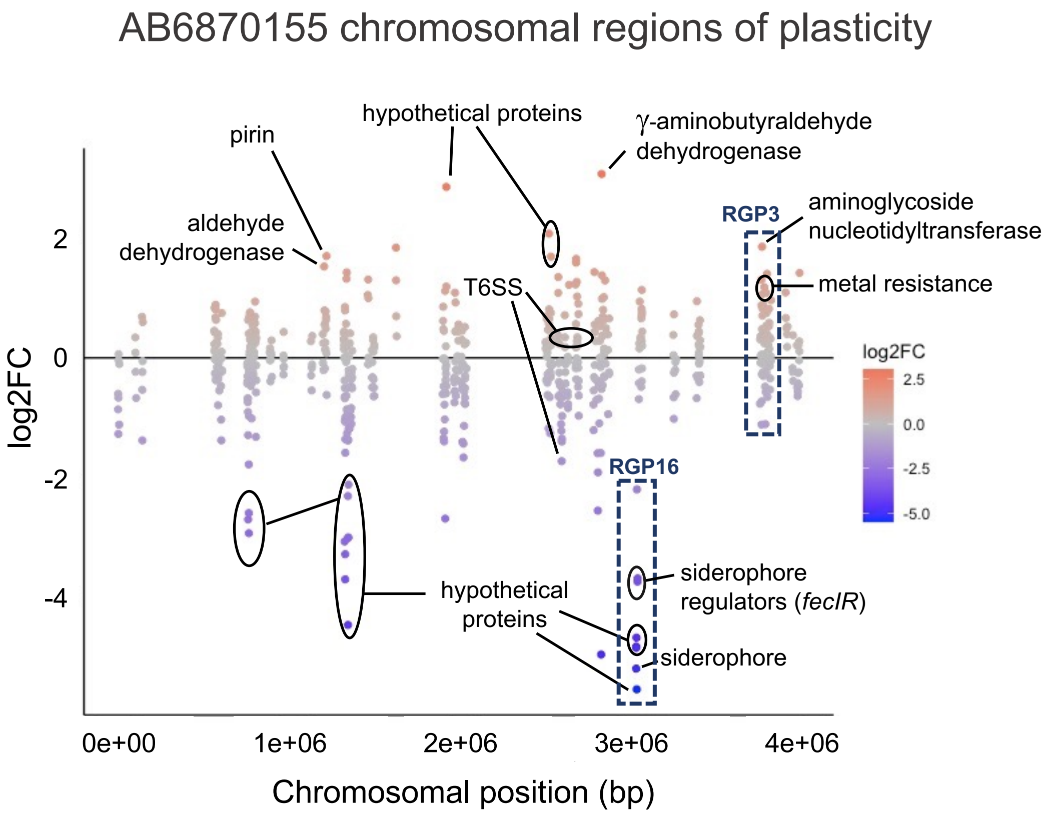

Figure S13 – AB6870155 chromosomal regions of genome plasticity (RGPs). Relative expression of AB6870155 pure culture grown biofilms versus AB6870155 + KP6870155 mixed-species grown biofilms presented as log2-fold change (log2FC) for genes located in RGPs computed by PPanGGOLiN [31]. Source data are provided as a Source Data file.

```
ACX60_15370       MFNNISQVLESIGFLSQHRSVYLQFSDASLNSQVFLQRIDGQHYLNQGMTAELICLSTNA   60
WP_000482858.1    MFNNIFQILESFGFLSQHRSVYLQFSDASLNSQVFLQRIDGQHYLNQGMTAELICLSTNA   60
MDBDAHJL_02760    MFNNIFQILESFGFLSQHRSVYLQFSDASLNSQVFLQRIDGQHYLNQGMTAELICLSTNA   60
ACICU_RS05745     MFNNIFQILESFGFLSQHRSVYLQFSDASLNSQVFLQRIDGQHYLNQGMTAELICLSTNA   60
                  ***** *:***:************************************************

ACX60_15370       HIPLKTFIGLQVAVDQVTDRGSFFRTTGIITGASQGQSDGALTLYKLAISDPTYLWHKRR   120
WP_000482858.1    HIPLKTFIGLQVAVDQVTDRGSFFRTTGIITGASQGQSDGALTLYKLTVSDPTYLWHKRR   120
MDBDAHJL_02760    HIPLKTFIGLQVAVDQVTDRGSFFRTTGIITGASQGQSDGALTLYKLTVSDPTYLWHKRR   120
ACICU_RS05745     HIPLKTFIGVQVAVDQVTDRGSFFRTTGIITGASQGQSDGALTLYKLAISDPTYLWHKRR   120
                  *********:*************************************::***********

ACX60_15370       NSRVFMNKSVKEISEILFQEWQGKSPLFASSLTLDLSGLKQTYDVRPFVMQLNESDYDFL   180
WP_000482858.1    NSRVFMNKSVKEISEILFQEWQGKSPLFASSLTLDLSGLKQTYDVRPFVMQLNESDYDFL   180
MDBDAHJL_02760    NSRVFMNKSVKEISEILFQEWQGKSPLFASSLTLDLSGLKQTYDVRPFVMQLNESDYDFL   180
ACICU_RS05745     NSRVFMNKSVKEISEILFQEWQGKSPLFASSLTLDLSGLKQTYDVRPFVMQLNESDYDFL   180
                  ************************************************************

ACX60_15370       TRLWRSEGISWLIDEAELTVASNTDNIQPQKLRLIDDNSQYQTLTRRTIRYHRSSATEQF   240
WP_000482858.1    TRLWRSEGISWLIDEAELTVASNMDNIQPQKLRLIDDNNQYQALTRRVIRYHRSSATEQF   240
MDBDAHJL_02760    TRLWRSEGISWLIDEAELTVASNMDNIQPQKLRLIDDNNQYQALTRRVIRYHRSSATEQF   240
ACICU_RS05745     TRLWRSEGISWLIDEAELTVASNMDNIQPQKLRLIDDNNQYQALTRRAIRYHRSSATEQF   240
                  *********************** **************.***:****.************

ACX60_15370       DSMTSLIAGRHLQPSSVFVQRWQSDVLQQTDGAGSVQSKHEHSSNYDNQSLNLEDAWHFS   300
WP_000482858.1    DSMTSLMADRSLQPTSIFVQRWQPDVLQQTDGAGSVQSKHQHSTNYDNQSLSLEEAWHFS   300
MDBDAHJL_02760    DSMTSLMADRSLQPTSIFVQRWQPDVLQQTDGAGSVQSKHQHSTNYDNQSLSLEEAWHFS   300
ACICU_RS05745     DSMTSLMADRSLQPTSIFVQRWQPDVLQQTDGAGSVQSKHQHSTNYDNQSLSLEEAWHFS   300
                  ******:*.* ***:*:****** ****************:**:*******.**:*****

ACX60_15370       PAWMQDLNGEDGATSASNQQLEKFNQNLSAYHDAQSKQFVAQTTVRDTQVGYWFELNEHP   360
WP_000482858.1    PAWMQDLNGEDGATSASNQQIEKFNQNLSAYYDAQSKQFIAKTTVRDTQVGYWFELNEHP   360
MDBDAHJL_02760    PAWMQDLNGEDGATSASNQQIEKFNQNLSAYYDAQSKQFIAKTTVRDTQVGYWFELNEHP   360
ACICU_RS05745     PAWMQDLNGEDGATSASNQQLEKFNQNLSAYYDAQSKQFIAKTTVRDTQVGYWFELNEHP   360
                  ********************:**********:*******:*:******************

ACX60_15370       EIDLHSGADKEFLIIGKHYYNQNNLPKDLDQQIQTLTTQSHWQSSHTKERQGNQLILQRR   420
WP_000482858.1    EIDQHESTDKEFLIIGKNYYNQNNLPKDLNQQIQTLLQQSDWQASNTDERQANQLILQRR   420
MDBDAHJL_02760    EIDQHESTDKEFLIIGKNYYNQNNLPKDLNQQIQTLLQQSDWQASNTDERQANQLILQRR   420
ACICU_RS05745     EIDQHESTDKEFLIIGKNYYNQNNLPKDLNQQIQTLVQQSDWQASNTDERQANQLILQRR   420
                  *** *..:*********:***********:******  **.**:*:*.***.********

ACX60_15370       HIKTVPEYQPLQDRPQASVQRARVVGPEGESIYVDQWGRIKVRFLFTRANDHSHDGGAGS   480
WP_000482858.1    YIPTTPAYNPQTHSPVAHPQRAKVVGPEGEEIYVDEWGRIKVRFLFTRSDDHSHDGGAGT   480
MDBDAHJL_02760    YIPTTPAYNPQTHSPVAHPQRAKVVGPEGEEIYVDEWGRIKVRFLFTRSDDHSHDGGAGT   480
ACICU_RS05745     YIPTTPAYNPQTHSPVAHPQRAKVVGPEGEEIYVDEWGRIKVRFLFTRSDDHSHDGGAGT   480
                  :* *.* *:*  . * *  ***:*******.****:************::*********:

ACX60_15370       NDNDTDSAWVDVLTPWAGAGYGARFLPRVGEIVVIDFFDGNIDRPFVVGRIHEAERHPAQ   540
WP_000482858.1    NNNDTDSAWIDVLTPWAGEGYGARFLPRIGEIVVIDFFNGDIDRPFVMGRIHEAQRHPTK   540
MDBDAHJL_02760    NNNDTDSAWIDVLTPWAGEGYGARFLPRIGEIVVIDFFNGDIDRPFVMGRIHEAQRHPTK   540
ACICU_RS05745     NNNDTDSAWIDVLTPWAGEGYGARFLPRIGEIVVINFFNGDIDRPFVMGRVHEAQRHPTK   540
                  *:*******:******** *********:******:**:*:******:**:***:***::

ACX60_15370       FDQKGQLPDTKKLSGIRSEEVDGKGFNQLRFDDTTGQISAQLQSSHAVSQLNLGNLSHPK   600
WP_000482858.1    FDNKGKLPDTKKLSGIRSKEVSGSGFGQLRFDDTPGQISTQLQSSHGASQLNLGKLSHPK   600
MDBDAHJL_02760    FDNKGKLPDTKKLSGIRSKEVSGSGFGQLRFDDTPGQISTQLQSSHGASQLNLGKLSHPK   600
ACICU_RS05745     FDNKGKLPDTKKLSGIRSKEVSGGGFGQLRFDDTPGQISTQLQSSHGASQLNLGKLSHPK   600
                  **:**:************:**.* **.******* ****:******..******:*****

ACX60_15370       DKAESDGRGEGFELRTDQWGAVRAGKGLLVSTHKQDQAQGTHLDAADAKQQIEGGLNNAK   660
WP_000482858.1    DKAESEDRGEGFELRTDQWGALRAGQGLLVSTHKQDNAKGDHLYAEVAKKQLEGSQTNSK   660
MDBDAHJL_02760    DKAESEDRGEGFELRTDQWGALRAGQGLLVSTHKQDNAKGDHLYAEVAKKQLEGSQTNSK   660
ACICU_RS05745     DKAESEDRGEGFELRTDQWGALRAGQGLLVSTHKQDNAKGDHLDAEVAKKQLEGSQTNSK   660
                  *****:.**************:***:**********:*:* ** *  **:*:**. .*:*

ACX60_15370       ALSEVAKNQQTDPLEMLENLKTFIEQIEEKDQDKAATFKQALMVLTAPNSIAVASNEDIH   720
WP_000482858.1    ALSDIAKNQKTDEIESIEQLKDFASQIQQ----QIAKFEKALLLLSSPDGIALSSSEDIH   716
MDBDAHJL_02760    ALSDIAKNQKTDEIESIEQLKDFASQIQQ----QIAKFEKALLLLSSPDGIALSSSEDIH   716
ACICU_RS05745     ALSDIAKNQKTDEIESIEQLKDFASQIQQ----QIAKFEKALLLLSSPDGIALSSSEDIH   716
                  ***::****:** :* :*:** * .**::    : *.*::**::*::*:.**::*.****

ACX60_15370       LSADGQLSQNAGDSISLSTQKNLIAHAQNKISLFAAQQGARLYAGKGKVEIQAQGDGADL   780
WP_000482858.1    ISADAQINQIAGDSINISTQKNVIAHAQNRLSLFAAQSGLKAVAAQGKVEIQAQADALDV   776
MDBDAHJL_02760    ISADAQINQIAGDSINISTQKNVIAHAQNRLSLFAAQSGLKAVAAQGKVEIQAQADALDV   776
ACICU_RS05745     ISADAQINQIAGDSINISTQKNVIAHAQNRLSLFAAQSGLKAVAAQGKVEIQAQADALDV   776
                  :***.*:.* *****.:*****:******::******.* :  *.:********.*. *:

ACX60_15370       IARKGIQIISTEDKIEINASKEIVITSGGSQIKINSSGIFPVTGGKFEVKAGQHVFMGGA   840
WP_000482858.1    LSKLGITISSTDDKVIISSPKEVKITGGSSQITLNGSGIFPKTGGKFQVNAGQHLFMGGA   836
MDBDAHJL_02760    LSKLGITISSTDDKVIISSPKEVKITGGSSQITLNGSGIFPKTGGKFQVNAGQHLFMGGA   836
ACICU_RS05745     LSKLGITISSTDDKVIISSPKEVKITGGSSQITLNGSGIFPKTGGKFQVNAGQHLFMGGA   836
                  ::: ** * **:**: *.: **: **.*.***.:*.***** *****:*:****:*****

ACX60_15370       KADYVLPSLPTQEIKTDDLLLEYLHSDGTPVKGADYEVLLSDGSIRKGKLDASGKAIVSG   900
WP_000482858.1    SANASAPELPKAKPMQGALELLRSYGGDNFFKQNSYKVIDSLGKQITGKLDGNGFAQVTG   896
MDBDAHJL_02760    SANASAPELPKAKPMQGALELLRSYGGDNFFKQNSYKVIDSLGKQITGKLDGNGFAQVTG   896
ACICU_RS05745     SANASAPELPKAKPMQGALELLRSYGGDNFFKQNSYKVIDSLGKQITGKLDGNGFAQVTG   896
                  .*:   *.**. :   . * *   :.... .*  .*:*: * *.  .****..* * *:*

ACX60_15370       VPAGRAKIQYGEDQSKDEFPALEVD---DWFTQLGSSTKTGKEE----------------   941
WP_000482858.1    IAPGPAKVVFEKDNTSAWLQSSDFKRNYTWAEPVKSVQGLMKNALEAVGQNTMSQLQNNL   956
MDBDAHJL_02760    IAPGPAKVVFEKDNTSAWLQSSDFKRNYTWAEPVKSVQGLMKNALEAVGQNTMSQLQNNL   956
ACICU_RS05745     IAPGPAKVVFEKDNTSAWLQSSDFKRNYTWAEPVKSVQGLMKNALEAVGQNTMSQLQNNL   956
                  :  * **: : :*::.  : : :..    *   : *     *:

ACX60_15370       ------------------------------------------------------------   941
WP_000482858.1    LSTDKNSFKNLGKNTLDNLAGQTVAQIKNQVTNTALNTVSK---QLNLNLSADQMKSLGQ   1013
MDBDAHJL_02760    LSTDKNSFKNLGKNTLDNLAGQTVAQIKNQVTNTALNTVSK---QLNLNLSADQMKSLGQ   1013
ACICU_RS05745     LSTDKNSFKT*VRIL*II*LAKQ*PRLKIR*LILPLILFQNNLISIYLPIK*KVLVKWRR   1010

ACX60_15370       ------------------------------------------------------------   941
WP_000482858.1    MATNPSQSLEMLK---------EQGGDFLSDQMTAKLFKTTNQESPIQQGDLDTFVRSKK   1064
MDBDAHJL_02760    MATNPSQSLEMLK---------EQGGDFLSDQMTAKLFKTTNQESPIQQGDLDTFVRSKK   1064
ACICU_RS05745     IQV---NLLKCLKSRAEIF*VIK*PQNYLRPQIKSHLFSRAI*I----------LLYGQK   1054

ACX60_15370       -   941
WP_000482858.1    -   1064
MDBDAHJL_02760    -   1064
ACICU_RS05745     S   1055
```

Figure S14 – AB6870155 vgrG1 gene alignment with *vgrG* genes in *A. baumannii* strains A85, ACICU and ATCC 17978. Clustal Omega multiple sequence alignment between *vgrG* genes in A85 (WP_000482858.1), ACICU (ACICU_RS06450) and ATCC17978 (ACX60_15370). DUF2345 is highlighted; SLFAAQ motif is indicated by red box.

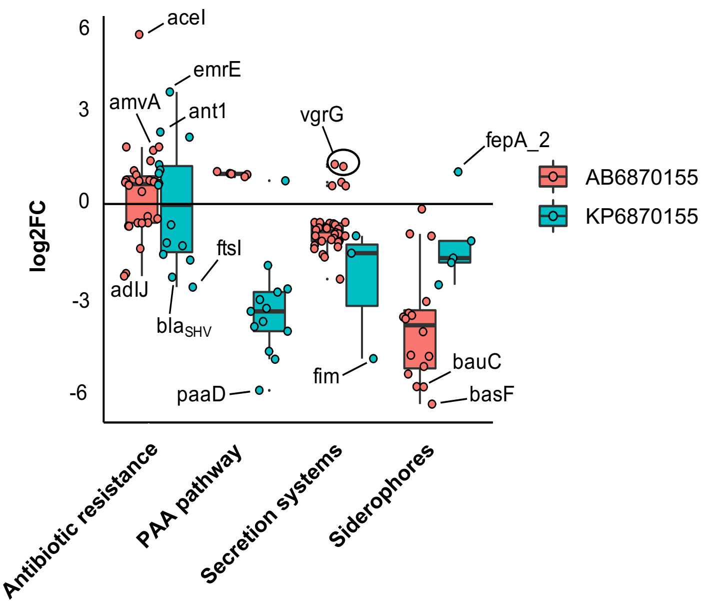

Figure S15 - RNA-seq gene expression of virulence genes in AB6870155 and KP6870155 co-cultures versus pure cultures of AB6870155 (pink) and versus pure cultures of KP6870155 (cyan) (n = 3). Boxes are bound by the first and third quartile with a horizontal line at the median and whiskers represent 1.5x the interquartile range. Source data are provided as a Source Data file.

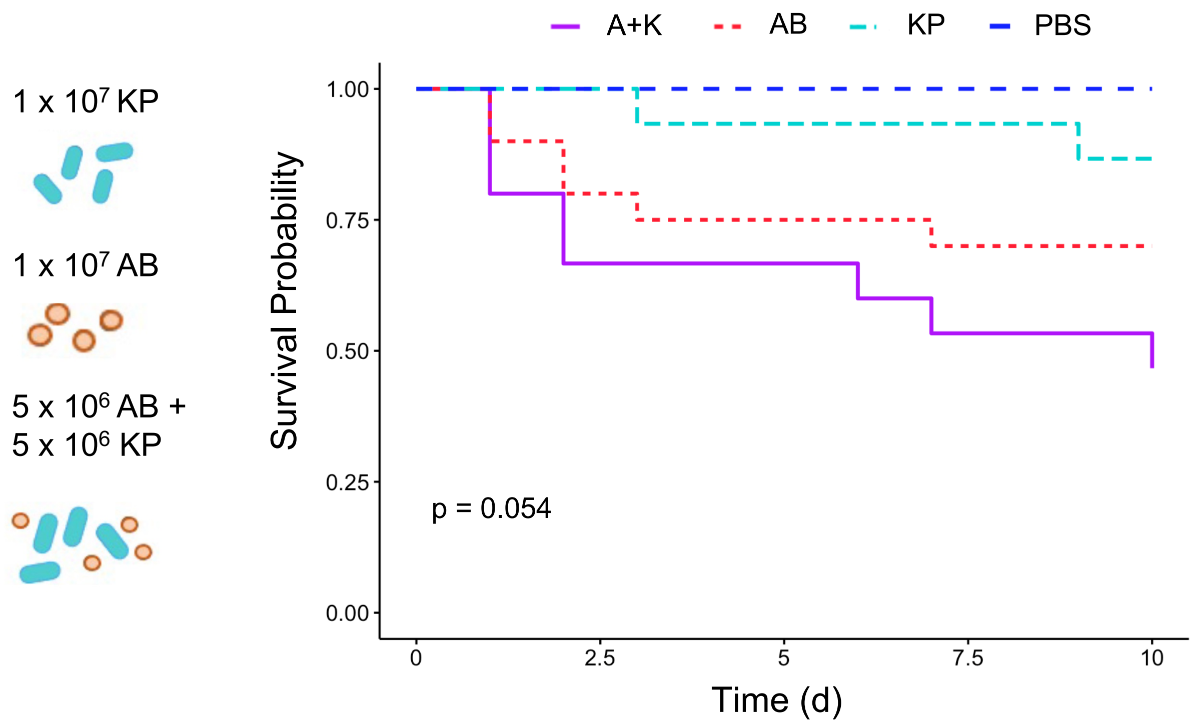

Figure S16 - Effect of inoculum dose of single infection versus coinfection with *A. baumannii* AB6870155 and *K. pneumoniae* KP6870155. Kaplan-Meier curves of single injected AB6870155 (AB) and KP6870155 (KP) and co-injected (A + K) at a 1:1 ratio in *G. mellonella* (p-value based on log-rank test). Source data are provided as a Source Data file.
