# Supplementary material for: Cross-protection and cross-feeding between Klebsiella pneumoniae and Acinetobacter baumannii promotes their co-existence
Source: Nat Commun. 2023 Feb 9;14:702. doi: 10.1038/s41467-023-36252-2 (PMC9911699; doi:10.1038/s41467-023-36252-2)
Supplement: Supplementary file 3 — Description of Additional Supplementary Files [file 41467_2023_36252_MOESM3_ESM.pdf]

Description of Additional Supplementary Files

File Name: Supplementary Data 1
Description: Regions of genome plasticity in *Klebsiella pneumoniae* complete genomes

File Name: Supplementary Data 2
Description: Antibiotic resistance genes in *K. pneumoniae* KP6870155 predicted by CARD

File Name: Supplementary Data 3
Description: *Acinetobacter baumannii* pangenome

File Name: Supplementary Data 4
Description: *Klebsiella pneumoniae* pangenome

File Name: Supplementary Data 5
Description: Regions of genome plasticity in *Acinetobacter baumannii* complete genomes

File Name: Supplementary Data 6
Description: Antibiotic resistance genes in *A. baumannii* AB6870155 predicted by CARD

File Name: Supplementary Data 7
Description: Expression profiles of shared genes between *A. baumannii* AB6870155 and *K. pneumoniae* KP6870155 in coculture vs. monoculture mapped to Biocyc pathways

File Name: Supplementary Data 8
Description: RNA-seq expression of *K. pneumoniae* KP6870155 coculture biofilms with *A. baumannii* AB6870155 in SLMM versus KP6870155 monoculture biofilms grown in SLMM

File Name: Supplementary Data 9
Description: Biolog PM01 and PM02 area under curve and growth status for *A. baumannii* AB6870155 and *K. pneumoniae* KP6870155

File Name: Supplementary Data 10
Description: RNA-seq expression of *A. baumannii* AB6870155 coculture biofilms with *K. pneumoniae* KP6870155 in SLMM versus AB6870155 monoculture biofilms grown in SLMM

File Name: Supplementary Data 11
Description: Iron-related genes in *A. baumannii* AB6870155 and *K. pneumoniae* KP6870155

File Name: Supplementary Data 12
Description: Minimum inhibitory concentrations for mono-cultures and co-cultures of *A. baumannii* (ATCC 17978 and E-072658) and *K. pneumoniae* KP6870155 (µg/mL)
