# Supplementary material for: Cross-protection and cross-feeding between Klebsiella pneumoniae and Acinetobacter baumannii promotes their co-existence
Source: Nat Commun. 2023 Feb 9;14:702. doi: 10.1038/s41467-023-36252-2 (PMC9911699; doi:10.1038/s41467-023-36252-2)
Supplement: Supplementary file 16 — Reporting Summary [file 41467_2023_36252_MOESM16_ESM.pdf]

nature portfolio

Corresponding author(s): Ian Paulsen, Karl Hassan

Last updated by author(s): Dec 21, 2022

Reporting Summary

Nature Portfolio wishes to improve the reproducibility of the work that we publish. This form provides structure for consistency and transparency in reporting. For further information on Nature Portfolio policies, see our Editorial Policies and the Editorial Policy Checklist.

Statistics

For all statistical analyses, confirm that the following items are present in the figure legend, table legend, main text, or Methods section.

n/a	Confirmed	
☐	☒	The exact sample size (*n*) for each experimental group/condition, given as a discrete number and unit of measurement
☐	☒	A statement on whether measurements were taken from distinct samples or whether the same sample was measured repeatedly
☐	☒	The statistical test(s) used AND whether they are one- or two-sided<br>*Only common tests should be described solely by name; describe more complex techniques in the Methods section.*
☒	☐	A description of all covariates tested
☒	☐	A description of any assumptions or corrections, such as tests of normality and adjustment for multiple comparisons
☐	☒	A full description of the statistical parameters including central tendency (e.g. means) or other basic estimates (e.g. regression coefficient) AND variation (e.g. standard deviation) or associated estimates of uncertainty (e.g. confidence intervals)
☐	☒	For null hypothesis testing, the test statistic (e.g. *F*, *t*, *r*) with confidence intervals, effect sizes, degrees of freedom and *P* value noted<br>*Give P values as exact values whenever suitable.*
☒	☐	For Bayesian analysis, information on the choice of priors and Markov chain Monte Carlo settings
☒	☐	For hierarchical and complex designs, identification of the appropriate level for tests and full reporting of outcomes
☒	☐	Estimates of effect sizes (e.g. Cohen's *d*, Pearson's *r*), indicating how they were calculated

Our web collection on statistics for biologists contains articles on many of the points above.

Software and code

Policy information about availability of computer code

Data collection

Data were collected as described in the manuscript.
Scanning electron microscopy (SEM) images were collected on the Phenom XL SEM (ThermoScientific)
Biofilm images were collected using the FV1000 CLSM (Olympus)
Genomes were sequenced with Oxford Nanopore long-read and Illumina sequencing (NextSeq 550) at the Microbial Genome Sequencing Center (MiGS)
Optical densities of cultures were collected with Fluorostar Omega spectrometer (BMG Labtech)
mRNA library preparation and Illumina NextSeq-500 sequencing were conducted in the Ramaciotti Centre for Genomics (UNSW, Australia)
HPLC data were collected on a Prominence UFLC system (Shimadzu, Japan), using an Aminex HPX-87H column (BioRad, USA)
Respiration and C-source (Biolog PM plates PM01-02) utilization data were collected on an Omnilog incubator/reader (Biolog)

Data analysis

-Genome annotation was performed with Prokka 1.14.6 using the -rfam parameter to find ncRNAs.
- pangenomes were constructed using PPanGGOLiN v 1.1.108 using the panrgp command to find regions of plasticity.
-SnapGene® software v 3.1.4 (from insightful Science; available at snapgene.com) was used to generate plasmid maps and PlasmidFinder was used to determine incompatibility groups.
-To find putative functions for proteins annotated as hypothetical, the eggNOG-mapper tool was used.
-Genomes were submitted to the Transporter Automated Analysis Pipeline (TransAAP; www.membranetransport.org) to identify predicted transporters.
-Needleman-Wuncsch pair-wise alignments were performed using ClustalW and multiple sequence alignments were performed using Clustal Omega.
-Iron-related genes were identified using FeGenie version 1 (November 2020) with the supplied HMM database.

-The Artemis genome browser was used to visualise the genomic regions containing siderophores to aid in constructing schematic representations of the genetic regions.
-Scripts and bioinformatics pipeline was uploaded to GitHub at https://github.com/amycainlab/coinfection_project.
-The K. pneumoniae KP6870155 assembled genome was submitted to Kleborate 30 using the --kaptive parameter to characterise its Klebsiella pneumoniae species complex, virulence loci, plasmid virulence loci, K capsule, O antigen (LPS) serotype.
-Identification of antibiotic resistance genes was performed using Comprehensive Antibiotic Resistance Database (CARD) v.2.0.3.
-Cell length measurements and statistical analysis of SEM data was performed using ImageJ software and the MicrobeJ plugin. Beeswarm plots representing this data were generated with beeswarm (v0.4.0)and Rserve (v1.8-10) R packages.
-CLSM biofilm images were processed using Imaris software (Bitplane).
-FastANI was used to calculate the average nucleotide identity (ANI) and alignment fractions (AF).
-distance matrix was generated with the parseDistanceMatrix function in R from the FastANI output. The ward.D2 method in the hclust R function was applied to the distance matrix and the output was converted to a dendrogram using the as.phylo() function using the R ape (v5.5) package.
-The growthcurver (v 0.3.1) R package was used to calculate the area under the logistic curve (auc_l). Scripts to generate plots were written in R (v.4.0.5) using the the ggplot2 package. Data processing and graphs were produced in RStudio using the tidyverse package (v1.3.1).
-RNA sequence reads were mapped to the respective hybrid assembled genomes using the STAR v 2.3.7a alignment tool with parameters set to --sjdbGTFfeatureExon CDS and --genomeSAindexNbases 8 for the genomeGenerate command and --alignIntronMax 1 for the mapping command.
-Samtools v 1.9 was used to sort and index alignment files which were subsequently input into featureCounts to generate counts tables.
-Read counts data was normalized and differential expression (DE) analysis conducted using the DESeq2 R package with ashr shrinkage estimator read counts of samples were normalized for sequencing depth and distortion caused by highly differentially expressed genes. A negative binomial model was used to test the significance of differential expression between two conditions. A cutoff of FDR (False Discovery Rate) of less than 0.05 and a log2 fold change >1.0 was used to determine significantly differentially expressed genes. Differential gene expression across various cellular pathways was visualized in Biocyc.
- Survival analysis for G. mellonella infection trials was performed using the Survminer (v 0.4.9) R package.

For manuscripts utilizing custom algorithms or software that are central to the research but not yet described in published literature, software must be made available to editors and reviewers. We strongly encourage code deposition in a community repository (e.g. GitHub). See the Nature Portfolio guidelines for submitting code & software for further information.

Data

Policy information about availability of data

All manuscripts must include a data availability statement. This statement should provide the following information, where applicable:

- Accession codes, unique identifiers, or web links for publicly available datasets
- A description of any restrictions on data availability
- For clinical datasets or third party data, please ensure that the statement adheres to our policy

Sequence reads were submitted to GenBank under project number PRJNA263680. A. baumannii AB6870155 genome BioSample accession SAMN03105183, K. pneumoniae KP6870155 genome BioSample accession SAMN23708555. RNA sequence reads were deposited in the NCBI Sequence Read Archive (SRA; http://www.ncbi.nlm.nih.gov/sra) under accession numbers SRR20379129, SRR20379130, SRR20379131, SRR20379132, SRR20379133, SRR20379134, SRR20379135, SRR20379136, SRR20379137. Source data are provided with this paper.
- The Comprehensive Antibiotic Resistance Database (CARD) v 2.0.3 was queried to identify antimicrobial resistance genes in the assembled genomes.

Human research participants

Policy information about studies involving human research participants and Sex and Gender in Research.

Reporting on sex and gender	*Use the terms sex (biological attribute) and gender (shaped by social and cultural circumstances) carefully in order to avoid confusing both terms. Indicate if findings apply to only one sex or gender; describe whether sex and gender were considered in study design whether sex and/or gender was determined based on self-reporting or assigned and methods used. Provide in the source data disaggregated sex and gender data where this information has been collected, and consent has been obtained for sharing of individual-level data; provide overall numbers in this Reporting Summary. Please state if this information has not been collected. Report sex- and gender-based analyses where performed, justify reasons for lack of sex- and gender-based analysis.*
Population characteristics	*Describe the covariate-relevant population characteristics of the human research participants (e.g. age, genotypic information, past and current diagnosis and treatment categories). If you filled out the behavioural & social sciences study design questions and have nothing to add here, write "See above."*
Recruitment	*Describe how participants were recruited. Outline any potential self-selection bias or other biases that may be present and how these are likely to impact results.*
Ethics oversight	*Identify the organization(s) that approved the study protocol.*

Note that full information on the approval of the study protocol must also be provided in the manuscript.

Field-specific reporting

Please select the one below that is the best fit for your research. If you are not sure, read the appropriate sections before making your selection.

☒ Life sciences ☐ Behavioural & social sciences ☐ Ecological, evolutionary & environmental sciences

For a reference copy of the document with all sections, see nature.com/documents/nr-reporting-summary-flat.pdf

Life sciences study design

All studies must disclose on these points even when the disclosure is negative.

Sample size	For in vivo studies with G. mellonella, the number of larvae (n =15) per group were appropriate for generating statistical significance. For cell culture experiments, no sample size calculation was performed and at least 3 independent replicates were used for each experiment as is the standard for cell culture experiments. Error bars represent the standard error or standard deviation (as indicated) for each experiment.
Data exclusions	Data were not excluded from the analysis.
Replication	All experimental data collected is from at least 3 biological replicates. All attempts at replication were successful and are reported as replicates.
Randomization	For in vivo G. mellonella experiments, larvae within a set weight range were allocated into random treatment groups. For all in vitro cell culture experiments this is not relevant as no subjective or hypothesis testing using clinical subjects was performed. For cell culture experiments, randomization does not apply as relevant.
Blinding	Blinding was not performed in the data collection or analysis phase of our study since no subjective measurements were made. The experimental work was carried out by seven different researchers for different experiments performed in the study.

Reporting for specific materials, systems and methods

We require information from authors about some types of materials, experimental systems and methods used in many studies. Here, indicate whether each material, system or method listed is relevant to your study. If you are not sure if a list item applies to your research, read the appropriate section before selecting a response.

Materials & experimental systems

n/a	Involved in the study	
☒	☐	Antibodies
☒	☐	Eukaryotic cell lines
☒	☐	Palaeontology and archaeology
☐	☒	Animals and other organisms
☒	☐	Clinical data
☒	☐	Dual use research of concern

Methods

n/a	Involved in the study	
☒	☐	ChIP-seq
☒	☐	Flow cytometry
☒	☐	MRI-based neuroimaging

Animals and other research organisms

Policy information about studies involving animals; ARRIVE guidelines recommended for reporting animal research, and Sex and Gender in Research

Laboratory animals	Galleria mellonella greater wax moth larvae in L5 stage between 180 - 220 mg in weight were incubated at 37C post-infection, with relative humidity at 70%, and light/dark cycle as follows (0.5h dawn - 5:30am-6am; 12h day - 6am-6pm; 0.5h dusk - 6-6:30pm; 11h night - 6:30pm-5:30am). Food was not provided post-injection.
Wild animals	This study did not involve wild animals.
Reporting on sex	Sex-based data was not included as sex is not considered in G. mellonella studies.
Field-collected samples	This study did not involve field-collected samples.
Ethics oversight	No ethical approval was required for working with Galleria mellonella as an in vivo.

Note that full information on the approval of the study protocol must also be provided in the manuscript.
